# Nasal Colonization of Humans with Occupational Exposure to Raw Meat and to Raw Meat Products with Methicillin-Susceptible and Methicillin-Resistant *Staphylococcus aureus*

**DOI:** 10.3390/toxins11040190

**Published:** 2019-03-30

**Authors:** Christiane Cuny, Franziska Layer, Sonja Hansen, Guido Werner, Wolfgang Witte

**Affiliations:** 1 Robert Koch Institute, Wernigerode Branch, 38855 Wernigerode, Germany; layerf@rki.de (F.L.); wernerg@rki.de (G.W.); EWWitte@t-online.de (W.W.); 2 Institute of Hygiene and Environmental Medicine, Charité, 12203 Berlin, Germany; sonja.hansen@charite.de

**Keywords:** MRSA, meat, occupational exposure

## Abstract

Livestock-associated methicillin-resistant *Staphylococcus aureus* (LA-MRSA) is widely disseminated as a nasal colonizer of conventionally raised livestock and of humans subjected to occupational exposure. Reports on contamination of raw meat raise the question as to whether occupationally exposed food handlers are at particular risk of nasal colonization by LA-MRSA. Here, we report the results from a cross-sectional study on nasal *S. aureus*/MRSA colonization of butchers, meat sellers, and cooks in Germany. We sampled 286 butchers and meat sellers in 26 butcheries and 319 cooks handling meat in 16 professional canteen kitchens. Swabs were processed on both blood agar plates and MRSA-selective plates. MRSA were confirmed by PCR for *mec* genes and by broth microdilution. All isolates were subjected to molecular typing. PCR for markers useful to differentiate human-adapted and animal-adapted subpopulations was performed due to the presence of clonal complexes known to occur in both livestock and humans (CC5, CC7, CC8, CC9, and CC398). Only two participants (0.33%) were colonized by MRSA (Hospital-associated MRSA ST22). Nasal colonization by methicillin-susceptible *S. aureus* (MSSA) was detected in 16.6% of cooks and in 26.2% of butchers and meat sellers. Among 16 of the isolates attributed to CC7, three were negative for the immune evasion gene cluster, suggesting an animal origin. Isolates attributed to CC5, CC8, and CC398 were negative for markers typical of animal-adapted subpopulations. The occupational handling of raw meat and raw meat products was not associated with nasal colonization by LA-MRSA.

## 1. Introduction

*Staphylococcus aureus* (*S. aureus*) is widely disseminated as a colonizer and as an infectious agent among humans and other animal hosts. The species can be subdivided into several clonal complexes by means of multi locus sequence tying. Some clonal complexes are mainly associated with particular host species, while others are less host specific (for review see Reference [1]). Human- and animal-adapted subpopulations differ by various genetic adaptations [2]. For example, the immune evasion gene cluster (IEC) is usually contained by human-adapted *S. aureus*, but it is mainly absent in the animal-adapted subpopulations [3]. Therefore, there are particular markers for the discrimination of human- and animal-adapted bacterial strains [4,5,6]. Livestock-associated MRSA (LA-MRSA) usually represent discrete subpopulations of clonal complexes of well-known human adapted subpopulations of *S. aureus* [2]. In particular, LA-MRSA CC398 (attributed to clonal complex CC398) has emerged in food production animals such as pigs, veal calves, and poultry worldwide (for review see [7]). Globally, the predominant lineages found in studies on LA-MRSA in swine populations are ST398 (most European [8] and some North American studies [9]), ST9 (most Asian studies [10]) and ST5 (North American studies [9]). It was scarcely surprising that LA-MRSA was also identified on retail meat of different animal origins (for review see [11]). A German study reported the contamination of 2.8% of pork end products with LA-MRSA CC398 [12], and it was subsequently detected in 32% of German turkey meat samples [13]. Furthermore, LA-MRSA CC398 was identified in the thawing liquid of 33% of broiler chicken carcasses [14]. Involvement of MSSA/MRSA CC398 in food intoxication has not been reported to date, and isolates attributed to this clonal complex only rarely contain enterotoxin genes [15]. Nasal colonization with LA-MRSA is frequent in humans with occupational livestock exposure (for review see [16]) and can persist after interruption of exposure [17,18]. Colonization of familial contacts of exposed persons is more rarely observed [19]. As known for *S. aureus* in general, humans colonized with LA-MRSA CC398 have an increased risk of developing various kinds of infections. LA-MRSA CC398 is not less virulent in humans than *S. aureus* in general [20]. More recently, colonization and infections of LA-MRSA CC398 were also reported for patients without any association of themselves or of their family members with livestock farming [21]. In addition to living in close vicinity to livestock farms from which LA-MRSA are exhausted, acquisition by handling contaminated meat and meat products should be taken into account. There are only a few studies that have addressed this question. MRSA colonization was identified in 1.15% of food handlers working in commercial kitchens in Hong Kong [22]. In the same city, 5.6% of butchers were colonized, of which 3% of them carried LA-MRSA ST9 [23]. Nasal MRSA colonization was not detected in a pilot study in the Netherlands involving 95 professional meat handlers [24]. Here, we report results from a cross-sectional study on nasal MRSA colonization of butchers and meat sellers working in butcheries and of cooks working in canteen kitchens. Methicillin-susceptible *S. aureus* (MSSA) isolates from the nasal swabs of handlers of both raw meat and raw meat products were also characterized in order to obtain a more general impression of the possibility of acquisition of *S. aureus* of animal origin.

## 2. Results

### 2.1. Characteristics of The Sample

Among the 605 persons who agreed to provide a nasal swab and to fill out a questionnaire on descriptive characteristics, 286 were butchers and meat sellers and 319 were cooks. The whole sample included 394 females (65.1%). A previous hospital stay was reported by 37 (6.1%) of the participants, previous prescription of antibiotics by 60 (9.9%), diabetes mellitus by 23 (3.8%), and superficial skin disorders by 27 (4.4%). Furthermore, 326 (53.9%) reported regular contact with companion animals. In addition to contact with companion animals (more frequent in the butchers and meat sellers’ groups) and hospital stay (more frequent in the cooks’ group), differences between both groups concerning the other variables were not significant (Table 1).

### 2.2. S. aureus Nasal Carriage

*S. aureus* was detected in 130 participants (21.5%; 95% confidence interval (CI) 18.2–24.8%). Carriage was significantly more frequent in males than in females (28.4%; 95% CI 22.2–35.1% versus 17.8%; 95% CI 14.2%–21.8%, *p* = 0.003, OR 1.83 [1.23–2.73]). Similar results were obtained when both occupational groups were analyzed separately (Table S1). To our surprise, colonization was significantly more frequently detected in butchers and meat sellers than in cooks (26.9%, CI 63–93% vs. 16.6%, CI 41–68%). There were, however, no significant differences in the frequency distribution of characteristics which might influence *S. aureus* nasal carriage (Table S1). Besides regular pet animal contact, the number of persons with diabetes mellitus, skin disorders, previous hospital stay, and antibiotic prescription was too low for statistical analysis of *S. aureus* carriage in both occupational groups. Differences in *S. aureus* carriage rates between persons with and without pet animal contact were not significant; therefore, the difference between both occupational groups was not influenced by this variable. 

### 2.3. Antibiotic Resistance

All of the 53 isolates obtained from 319 cooks and 75 of the 77 isolates obtained from 286 butchers and meat sellers were phenotypically susceptible to oxacillin and cefoxitin and negative for *mecA*. Seven of these isolates exhibiting an MIC of 1.0 mg/L for oxacillin were negative for *mecC* by PCR. Among the 77 isolates obtained from butchers and meat sellers, only two exhibited resistance to oxacillin and cefoxitin. Both MRSA isolates contained *mecA* on SCC*mec*IV and exhibited *spa*-type t032, which is typical for hospital-associated MRSA ST22. Altogether MRSA was detected in two participants (0.33%; 95% CI 0–1.1%) that were both females who had worked as meat sellers and had an age range of 41–50 years, with no report of a previous hospital stay, a previous antibiotic prescription, diabetes mellitus, or skin disorder. The phenotypical antibiotic resistance patterns of the 130 isolates investigated are shown in Table 2. Among the isolates, 43% were susceptible to all antibiotics tested. Amongst the MSSA, resistance to penicillin was most common (50%), followed by resistance to erythromycin (7.8%). Resistance to both penicillin and erythromycin was found in seven isolates, three of which (*spa*-types t571 and t1451) were attributed to CC398. There is a limitation with respect to no checking erythromycin resistant isolates for the presence of the inducible macrolide-lincosamide-streptogramin B phenotype.

### 2.4. Results from Typing

As shown in Table 3, the isolates exhibited a total of 52 *spa*-types. Based on related repeat patterns, the observed *spa-*types were grouped into 6 *spa*-clonal complexes (*spa*-CCs). Eleven *spa*-types were singletons (repeat pattern not associated with the patterns of other isolates from this study), four revealed as non-typeable, and one was excluded because it contained less than five repeats. The isolates were attributed to 14 MLST-based clonal complexes (CCs). *Spa*-types t084 (*n* = 14), t091 (*n* = 14), and t008 (*n* = 9) were particularly frequent. Because of related *spa*-repeat patterns, isolates attributed to MLST-sequence type clonal complexes CC7 and CC15 are grouped in *spa*-CC spa-CC084/346. *Spa*-CC045 usually includes *S. aureus* isolates attributed to CC15 by MLST. Attribution to CCs for isolates from both occupational groups of carriers is shown in Table S2. Subpopulations adapted to humans as well as to animals are known for CCs 5, 8, 9, and 398, and they can be discriminated by PCR as described in the materials and methods section. In addition*, S. aureus* CC7 was observed in humans as well as in chicken [25]. Among the 16 isolates attributed to CC7, three were negative for IEC which suggests an animal origin. All of the seven isolates attributed to clonal complex 5 were negative for pathogenicity island SaPIAv, and all of the 13 isolates attributed to CC8 were negative for *lukM* and for seo141, respectively, suggesting no animal origin. The four isolates attributed to CC398 were assigned to the human-adapted subpopulation (Table S3).

## 3. Discussion

The *S. aureus* carriage rate found in our cross-sectional study for butchers and meat sellers roughly corresponds to results from recent studies in Central, North Western, and Northern Eastern Germany [26,27,28]. The carriage rate was higher in men in both occupational groups. This corresponds to results from previous German studies [26,27] and to observations in different countries [29]. The reasons for the surprisingly lower carriage rate in cooks remain unclear. Both occupational groups did not differ significantly by gender distribution nor to other conditions which are known to predispose to nasal carriage of *S. aureus*, such as diabetes mellitus and superficial skin disorders [29]. *S. aureus* carriage rates seem to decrease with age [30]. However, there were no significant differences in age group distribution between both occupational groups. Thus, we cannot exclude an influence of the work habit. Inhalation of antibacterial compounds that evaporate from the cleanser at the end of the working day when warm stoves are cleaned might suppress colonization. Another explanation would be that cooks more frequently wash their hands while on duty and might get used to this in general. Hands seem to play an important role in human to human transmission via contamination of surfaces [31,32]. Furthermore, we have to consider that although sampling only once for each person captures persistent as well as transient colonization, there are also intervals with no demonstration of *S. aureus* in persistent carriers [26,33].

Carriage of MRSA was found in two persons from the butcher’s group (0.7%). This observation corresponds to data from recent population-based studies in Germany [26,27,34]. Both MRSA isolates from butchers in this study were attributed to clonal lineage ST22, a hospital-associated epidemic MRSA lineage which is disseminated worldwide [35]. It is particularly abundant in Germany where it accounts for 84.4% of all MRSA isolates from wound infections and bacteremia [36]. Emergence of HA-MRSA ST22 in the community was also reported from Portugal [37]. In contrast to reports from Hong Kong, LA-MRSA colonization was not observed in humans with occupational exposure to raw meat and raw meat products in our study. In addition to different working habits, this might be due to different capacities of LA-MRSA CC9 and LA-MRSA CC398 for human nasal colonization. Furthermore, the low quantities of MRSA on retail meat reported so far [24,38,39] may not be sufficient for acquisition of nasal colonization by handling these products. On the other hand, an epidemiological study in the Netherlands identified regular consumption of poultry as a risk factor for acquisition of nasal MRSA colonization [40].

The frequency of resistance to penicillin among the MSSA corresponded to data from previous studies on MSSA from nasal colonization in Germany [27,28]. Resistance to erythromycin was slightly more frequent and corresponded to data reported for MSSA from infections in the German community [41]. The frequency to tetracycline was low. Tetracycline resistance is usually prevalent in MSSA and MRSA from livestock, and the same applies to erythromycin resistance (for European studies [42,43,44]).

Particular CCs were abundant among the MSSA, including CC15 and CC22, followed by CCs 7, 8, and 45. Besides CC7, this corresponds to data from a previous study in North-Eastern Germany [28]. However, isolates attributed to CC25 were also prevalent in this region but absent among the isolates from the study presented here. These observations suggest regional differences in the population structure of *S. aureus* colonizing humans. Colonization with MSSA of probable animal origin was also rare; only three isolates attributed to ST7 were negative for IEC. *S. aureus* CC7 is not only prevalent in human colonization but also identified from chicken meat [25].

In summary, our findings suggest that the risk of LA-MRSA colonization from handling meat seems to be very low for professionals working in canteen kitchens and butcheries. Most probably, the general population may be at an even lower risk, good kitchen hygiene practice provided.

## 4. Materials and Methods 

### 4.1. Study Participants 

In Germany, food-workers are not routinely screened for *S. aureus* carriage. Mediated by the management of butcheries and canteen kitchens, butchers, meat sellers, and cooks were contacted for voluntary participation in the study by an information letter. In case of agreement and return of a declaration of consent they obtained swabs for self-collection of nasal swabs, an instruction for self-collection, and a short questionnaire asking for information on basic demographic characteristics such as age, sex, and potential risk factors for colonization with methicillin-sensitive and methicillin-resistant *S. aureus* (MSSA and MRSA). Feasibility of self-collection was reported previously [45]. A total of 286 butchers and meat sellers from 26 butcher’s shops as well as 319 cooks from 16 canteen kitchens were enrolled in the study. Companies participating were located in federal states Saxony-Anhalt, Lower Saxony, Saxony, North-Rhine-Westphalia, and Thuringia. The study was approved by the ethical committee of the medical faculty of Magdeburg University (#33/14).

### 4.2. Sampling and Primary Diagnostics

For taking nasal swabs, the transystem^®^ from Hain- Lifescienes, 72,147 Nehren, Germany, was used. Both nostrils of each participant were sampled by the same swab. After non-selective enrichment in cation-adjusted Mueller–Hinton broth, aliquots were streaked on CHROMagar™ MRSA from Becton Dickinson (Heidelberg, Germany) and in parallel on Mueller–Hinton blood agar plates (Oxoid, Wesel, Germany). After incubation at 37 °C for 24 h, one suspicious colony was subcultured on sheep blood agar, except differences in colonial morphology and haemolysis. Confirmation of *S. aureus* was performed by demonstration of the clumping factor and additionally by the tube coagulase test. For the detection of the clumping factor, we used a solution of fibrinogen from human plasma (Sigma-Aldrich, Taufkirchen, Germany) of 2 mg/mL 0.85% NaCl). For the tube coagulase test, we used fresh ready to use human plasma provided by DRK blood donation service, 31,831 Springe, Germany. In the case of negative results, we performed PCR for the *S. aureus* specific region of *tuf* by use of primers and PCR conditions according to Reference [46]. For PCR, genomic DNA was extracted from *S. aureus* grown in nutrient broth by use of the DNeasy tissue kit (Qiagen, Hilden, Germany), and lysostaphin (100 mg/L; Sigma, Taufkirchen, Germany) for bacterial lysis.

### 4.3. Typing and Further Characterization

All *S. aureus* isolates were subjected to *spa* typing as described previously [47]. Related spa types (costs ≤ 4) were grouped into *spa*-clonal clusters (*spa* CC) using the BURP algorithm (Ridom StaphType software version 2.2.1, RidomGmbH, Würzburg, Germany). *Spa-*CCs were allocated to MLST CCs through the SpaServer database (www.spaserver.ridom.de) and the database of the German National Reference Center for Staphylococci and Enterococci. For a subset of isolates, MLST was performed as described [48]. Antimicrobial susceptibility testing was performed by using the broth microdilution method and applying EUCAST breakpoints (version 8.1), for interpretation of the results (www.eucast.org). Test panels were from own production. The following antibiotics were tested: penicillin, oxacillin, cefoxitin, fosfomycin, gentamicin, linezolid, erythromycin, clindamycin, tetracycline, tigecycline, vancomycin, teicoplanin, ciprofloxacin, mupirocin, moxifloxacin, daptomycin, fusidic acid-sodium, rifampicin, and trimethoprim/sulfamethoxazole. 

PCR for *mec*A, *mec*C, and SCC*mec* was performed as described previously [19,49]. Markers for attribution of isolates to human- and animal adapted subpopulations of particular clonal lineages were detected by PCR as described below.

PCR for IEC (*sak, chp, scn, sea, sep*) was performed for isolates attributed to CC7 and CC9 as described previously [47]. The avian subpopulation of CC5 contains pathogenicity island SaPIAv [4], and primers SAPIAvF1/R1 for detection were used as described [4]. There is obviously a bovine subpopulation in CC8 which contains *luk*M [5] as known for clonal lineages typically associated with ruminants [50]. Primers used for detection correspond to Reference [51]. Furthermore, the bovine subpopulation of ST8 harbors nucleotide sequence of 26 in SCCM186 [5], a homologue of SEO-141 in *S. epidermidis* ATCC12228. Primers used for detection were 5′-ATGGGAATCAAAGACGTA and 5′CAATTGTTATTATCCTGCTGTC (134366-134383 and 134484-13458, respectively in NC_004461). For CC398, an ancestral human-adapted and an animal-adapted subpopulation are known. Rapid discrimination was performed by singleplex PCR for the canonical single nucleotide polymorphism in the SAPIG_2511 locus (nucleotide 2,597,585 in AM990992 vs. nucleotide 2,440,348 in CP0003045), according to published protocols [42]. The cycling scheme for these PCR reactions was: 95 °C, 300s [95 °C, 30 s; 55 °C, 30 s; 72 °C, 30 s] × 35; 72 °C, 240 s. PuReTaq Ready-To-Go PCR Beads, GE Healthcare, were used.

### 4.4. Statistical Analysis 

A two-tailed *t*-test and Fishers exact test (by means of EpiInfoTM, version 7.2.2.6, from CDC, Atlanta, GA, USA) were used for calculation of p and OR.

## Figures and Tables

**Table 1:** Descriptive characteristics of the study participants.

			All (*n* = 605)		Butchers, Meat Sellers (*n* = 286)		Cooks (*n* = 319)		*p* ^1^
Sex		female	394	65.1%	195	68.2%	199	62.4%	
		male	211	34.8%	91	31.8%	120	37.6%	0.158
Age groups		<20	12	2%	9	3.1%	3	0.9%	0.0986
		21–30	81	13.4%	33	11.50%	48	15.0%	0.211
		31–40	116	19.2%	41	14.30%	75	23.5%	0.058
		41–50	150	24.8%	80	30.0%	70	22.0%	0.169
		51–60	182	30.0%	92	32.2%	90	28.2%	0.313
		>61	64	10.6%	31	10.8%	33	10.3%	0.042
Hospital stay			37	6.1%	8	2.8%	29	9.0%	0.022
Antibiotic prescription			60	9.9%	22	7.6%	38	11.0%	0.11
Diabetes mellitus			23	3.8%	12	4.2%	11	3.4%	0.789
Skin disorders			27	4.5%	19	6.6%	8	2.5%	0.023
Pet animal contact			326	53.90%	176	61.5%	150	47.0%	0.0004

^1^ corrected p calculated by a two tailed *t*-test.

**Table 2:** Antibiotic resistance patterns of *S. aureus* (*n* = 130) isolated from nasal swabs of cooks, butchers, and meat sellers.

Antibiotic Resistance Phenotypes	Frequency	Resistance to Singular Antibiotics in MSSA (*n* = 128)	Frequency
Susceptible	56 (43.0%)		
PEN	55 (42.3%)	PEN	64 (50.0%)
PEN, ERY	7 (5.4%)	ERY	13 (10.1%)
PEN, TET	1 (0.77%)	CLI	1 (0.8%)
PEN, CIP	1 (0.77%)	TET	2 (1.6%)
PEN, OXA, CIP, MFL	1 (0.77%)	CIP	3 (2.3%)
PEN, OXA, ERY, CLI, CIP	1 (0.77%)		
ERY	5 (3.9%)		
TET	1 (0.77%)		
CIP	2 (0.15%)		

Abbreviations: CIP = ciprofloxacin, CLI = clindamycin, ERY = erythromycin, MFL = moxifloxacin, OXA = oxacillin, PEN = benzylpenicillin, TET = tetracycline. Antibiotics tested: penicillin, oxacillin, cefoxitin, fosfomycin, gentamicin, linezolid, erythromycin, clindamycin, tetracycline, tigecycline, vancomycin, teicoplanin, ciprofloxacin, mupirocin, moxifloxacin, daptomycin, fusidic acid-sodium, rifampicin, and trimethoprim/sulfamethoxazole.

**Table 3:** *Spa*-types and *spa*-clonal complexes of methicillin-susceptible *S. aureus* (MSSA) and methicillin-resistant *S. aureus* (MRSA).

*spa*-CC	*spa*-Types (No. Isolates)	No. Isolates	Attribution to CC (ST)
Singleton	t127(3)	3	CC1 3 (2.3%)
*spa*-CC002	t002(5), t1265(1), t1794(1)	7	CC5 7 (5.4%)
*spa*-CC084/346	t091(14), t1943(1)	15	CC7 16 (12.3%)
Non typeable	t2932(1)	1	
*spa-*CC121	t008(9), t121(2), t190(1), t292(1)	13	CC8 13 (10.0%)
Singleton	t209(1)	1	CC9 1 (0.8%)
*spa*-CC084/346	t084(14), t346(4), t499(2), t491(1)	21	CC15 25 (19.2%)
Non typeable	15546(1), t15664(1), t15712(1)	3	
Excluded	t5497(1)	1	
*spa*-CC005	t005(12), t006(1), t032(2) ^1^, t223(1) t310(1), t449(1), t17201(1)	19	CC22 21 (16.2%)
Singletons	t417(1), t420(1)	2	
*spA*-CC012	t012(6), t018(2), t021(2), t122(1), t253(1), t338(1), t789(1)	14	CC30 15 (11.5%)
Singleton	t1827(1)	1	
No founder	t136(1), t166(1)	2	CC34 4 (3.0%)
Singleton	t089(2)	2	
*spa*-CC015	t015(7), t073(4), t331(1), t505(1), t15726(1)	14	CC45 16 (12.3%)
Singletons	t004(1), t1460(1)	2	
Singleton	t056(2)	2	CC101 2 (1.5%)
Singleton	t159(2)	2	CC121 2 (1.5%)
Singleton	t493(1)	1	ST182 1 (0.8%)
No founder	t571(2), t1451(2)	4	CC398 4 (3.0%)
Total		130	

^1^ Both isolates were MRSA.

## Supplementary Materials

The following are available online at https://www.mdpi.com/2072-6651/11/4/190/s1, Table S1: Prevalence and factors associated with nasal *S. aureus* carriage, Table S2: Distribution of *spa*-types and clonal complexes among *S. aureus* from butchers and meat sellers, and from cooks, Table S3: Results from PCR for attribution to animal adapted subpopulations.

## References

[1] Cuny C., Friedrich A., Kozytska S., Layer F., Nübel U., Ohlsen K., Strommenger B., Walther B., Wieler L., Witte W. (2010). Emergence of methicillin-resistant *Staphylococcus aureus* (MRSA) in different animal species. Int. J. Med. Microbiol..

[2] Fitzgerald J.R. (2012). Livestock-associated *Staphylococcus aureus*: Origin, evolution and public health threat. Trends Microbiol..

[3] Sung J.M., Lloyd D.H., Lindsay J.A. (2008). *Staphylococcus aureus* host specificity: Comparative genomics of human versus animal isolates by multistrain microarray. Microbiology.

[4] Lowder B.V., Guinane C.M., Ben Zakour N.L., Weinert L.A., Conway-Morris A., Cartwright R.A., Simpson A.J., Rambaut A., Nübel U., Fitzgerald J.R. (2009). Recent human-to-poultry host jump, adaptation, and pandemic spread of *Staphylococcus aureus*. Proc. Natl. Acad. Sci. USA.

[5] Resch G., François P., Morisset D., Stojanov M., Bonetti E.J., Schrenzel J., Sakwinska O., Moreillon P. (2013). Human-to-bovine jump of *Staphylococcus aureus* CC8 is associated with the loss of a β-hemolysin converting prophage and the acquisition of a new staphylococcal cassette chromosome. PLoS ONE.

[6] Stegger M., Liu C.M., Larsen J., Soldanova K., Aziz M., Contente-Cuomo T., Petersen A., Vandendriessche S., Jiménez J.N., Mammina C. (2013). Rapid differentiation between livestock-associated and livestock-independent *Staphylococcus aureus* CC398 clades. PLoS ONE.

[7] Cuny C., Wieler L.H., Witte W. (2015). Livestock-Associated MRSA: The impact on humans. Antibiotics.

[8] Pantosti A. (2012). Methicillin-Resistant *Staphylococcus aureus* associated with animals and Its relevance to human health. Front. Microbiol..

[9] Smith T.C., Thapaliya D., Bhatta S., Mackey S., Engohang-Ndong J., Carrel M. (2018). Geographic distribution of livestock-associated Staphylococcus aureus in the United States. Microbes Infect..

[10] Chuang Y.Y., Huang Y.C. (2015). Livestock-associated meticillin-resistant *Staphylococcus aureus* in Asia: An emerging issue?. Int. J. Antimicrob. Agents..

[11] Kluytmans J.A. (2010). Methicillin-resistant *Staphylococcus aureus* in food products: Cause for concern or case for complacency?. Clin. Microbiol. Infect..

[12] Beneke B., Klees S., Stührenberg B.A., Kraushaar B., Tenhagen B.A. (2011). Prevalence of methicillin-resistant *Staphylococcus aureus* in a fresh meat pork production chain. J. Food Prot..

[13] Vossenkuhl B., Brandt J., Fetsch A., Käsbohrer A., Kraushaar B., Alt K., Tenhagen B.A. (2014). Comparison of spa types, SCC*mec* types and antimicrobial resistance profiles of MRSA Isolated from turkeys at farm, Ssaughter and from retail meat indicates transmission along the production chain. PLoS ONE.

[14] Cuny C., Layer F., Witte W. (2011). *Staphylococcus aureus* and MRSA in thawing liquid of broiler chicken carcasses and their relation to clonal lineages from humans. Intern. J. Med. Microbiol..

[15] Argudín M.A., Tenhagen B.A., Fetsch A., Sachsenröder J., Käsbohrer A., Schroeter A., Hammerl J.A., Hertwig S., Helmuth R., Bräunig J. (2011). Virulence and resistance determinants of German *Staphylococcus aureus* ST398 isolates from nonhuman sources. Appl. Environ. Microbiol..

[16] Klous G., Huss A., Heederik D.J., Coutinho R.A. (2016). Human-livestock contacts and their relationship to transmission of zoonotic pathogens, a systematic review of literature. One Health.

[17] Köck R., Loth B., Köksal M., Schulte-Wülwer J., Harlizius J., Friedrich A.W. (2012). Persistence of nasal colonization with livestock-associated methicillin-resistant *Staphylococcus aureus* in pig farmers after holidays from pig exposure. Appl. Environ. Microbiol..

[18] Walter J., Espelage W., Adlhoch C., Cuny C., Schink S., Jansen A., Witte W., Eckmanns T., Hermes J. (2017). Persistence of nasal colonisation with methicillin resistant *Staphylococcus aureus* CC398 among participants of veterinary conferences and occurrence among their household members: A prospective cohort study, Germany 2008-2014. Vet. Microbiol..

[19] Cuny C., Nathaus R., Layer F., Strommenger B., Altmann D., Witte W. (2009). Nasal colonization of humans with methicillin-resistant *Staphylococcus aureus* (MRSA) CC398 with and without exposure to pigs. PLoS ONE.

[20] Becker K., Ballhausen B., Kahl B.C., Köck R. (2017). The clinical impact of livestock-associated methicillin-resistant *Staphylococcus aureus* of the clonal complex 398 for humans. Vet. Microbiol..

[21] Deiters C., Günnewig V., Friedrich A.W., Mellmann A., Köck R. (2015). Are cases of Methicillin-resistant *Staphylococcus aureus* clonal complex (CC) 398 among humans still livestock-associated?. Int. J. Med. Microbiol..

[22] Ho J., O’Donoghue M.M., Boost M.V. (2014). Occupational exposure to raw meat: A newly-recognized risk factor for *Staphylococcus aureus* nasal colonization amongst food handlers. Int. J. Hyg. Environ. Health.

[23] Boost M., Ho J., Guardabassi L., O’Donoghue M. (2013). Colonization of butchers with livestock-associated methicillin-resistant *Staphylococcus aureus*. Zoonoses Public Health.

[24] De Jonge R., Verdier J.E., Havelaar A.H. (2010). Prevalence of methicillin-resistant *Staphylococcus aureus* amongst professional meat handlers in the Netherlands, March-July 2008. Euro Surveill..

[25] Krupa P., Bystroń J., Bania J., Podkowik M., Empel J., Mroczkowska A. (2014). Genotypes and oxacillin resistance of *Staphylococcus aureus* from chicken and chicken meat in Poland. Poult. Sci..

[26] Becker K., Schaumburg F., Fegeler C., Friedrich A.W., Köck R. (2017). *Staphylococcus aureus* from the German general population is highly diverse. Int. J. Med. Microbiol..

[27] Mehraj J., Akmatov M.K., Strömpl J., Gatzemeier A., Layer F., Werner G., Pieper D.H., Medina E., Witte W., Pessler F. (2014). Methicillin-sensitive and methicillin-resistant *Staphylococcus aureus* nasal carriage in a random sample of non-hospitalized adult population in northern Germany. PLoS ONE.

[28] Holtfreter S., Grumann D., Balau V., Barwich A., Kolata J., Goehler A., Weiss S., Holtfreter B., Bauerfeind S.S., Döring P. (2016). Molecular Epidemiology of *Staphylococcus aureus* in the General Population in Northeast Germany: Results of the Study of Health in Pomerania (SHIP-TREND-0). J. Clin. Microbiol..

[29] Sollid J.U., Furberg A.S., Hanssen A.M., Johannessen M. (2014). *Staphylococcus aureus*: Determinants of human carriage. Infect. Genet. Evol..

[30] Sakr A., Brégeon F., Mège J.L., Rolain J.M., Blin O. (2018). *Staphylococcus aureus* Nasal Colonization: An Update on Mechanisms, Epidemiology, Risk Factors, and Subsequent Infections. Front. Microbiol..

[31] Wertheim H.F., Melles D.C., Vos M.C., van Leeuwen W., van Belkum A., Verbrugh H.A., Nouwen J.L. (2005). The role of nasal carriage in *Staphylococcus aureus* infections. Lancet Infect. Dis..

[32] Ho J., Boost M.V., O’Donoghue M.M. (2015). Tracking sources of *Staphylococcus aureus* hand contamination in food handlers by spa typing. Am. J. Infect. Control..

[33] Miller R.R., Walker A.S., Godwin H., Fung R., Votintseva A., Bowden R. (2014). Dynamics of acquisition and loss of carriage of *Staphylococcus aureus* strains in the community: The effect of clonal complex. J. Infect..

[34] Köck R., Werner P., Friedrich A.W., Fegeler C., Becker K. (2015). Prevalence of Multiresistant Microorganisms (PMM) Study Group. New Microbes New Infect..

[35] Holden M.T., Hsu L.Y., Kurt K., Weinert L.A., Mather A.E., Harris S.R., Strommenger B., Layer F., Witte W., de Lencastre H. (2013). A genomic portrait of the emergence, evolution, and global spread of a methicillin-resistant *Staphylococcus aureus* pandemic. Genome Res..

[36] Layer F., Stromenger B., Cuny C., Werner G. (2015). Eigenschaften, Häufigkeit und Verbreitung von MRSA in Deutschland—Update 2013/2014. Epidemiol. Bull..

[37] Tavares A., Miragaia M., Rolo J., Coelho C., de Lencastre H. (2013). High prevalence of hospital-associated methicillin-resistant *Staphylococcus aureus* in the community in Portugal: Evidence for the blurring of community-hospital boundaries. Eur. J. Clin. Microbiol. Infect. Dis..

[38] Weese J., Avery B., Reid Smith R. (2010). Detection and quantification of methicillin resistant *Staphylococcus aureus* (MRSA) clones in retail meat products. Lett. Appl. Microbiol..

[39] Thapaliya D., Forshey B.M., Kadariya J., Quick M.K., Farina S., O’ Brien A., Nair R., Nworie A., Hanson B., Kates A. (2017). Prevalence and molecular characterization of Staphylococcus aureus in commercially available meat over a one-year period in Iowa, USA. Food Microbiol..

[40] Van Rijen M.M., Kluytmans-van den Bergh M.F., Verkade E.J., Ten Ham P.B., Feingold B.J., Kluytmans J.A., CAM Study Group (2013). Lifestyle-Associated Risk Factors for Community-Acquired Methicillin-Resistant *Staphylococcus aureus* Carriage in the Netherlands: An Exploratory Hospital-Based Case-Control Study. PLoS ONE.

[41] ARS—Antibiotika-Resistenz-Surveillance. https://ars.rki.de.

[42] Armand-Lefevre L., Ruimy R., Andremont A. (2005). Clonal comparison of *Staphylococcus aureus* isolates from healthy pig-farmers, human controls, and pigs. Emerg. Infect. Dis..

[43] Van der Wolf P.J., Rothkamp A., Junker K., de Neeling A.J. (2012). *Staphylococcus aureus* (MSSA) and MRSA (CC398) isolated from post-mortem samples from pigs. Vet. Microbiol..

[44] Nemati M., Hermans K., Lipinska U., Denis O., Deplano A., Struelens M., Devriese L., Pasmans F., Haesebrouck F. (2008). Antimicrobial resistance of old and recent Staphylococcus aureus isolates from poultry: First detection of livestock-associated methicillin-resistant strain ST398. Antimicrob. Agents Chemother..

[45] Akmatov M.K., Mehraj J., Gatzemeier A., Strömp J., Witte W., Krause G., Pessler F. (2014). Serial home-based self-collection of anterior nasal swabs to detect *Staphylococcus aureus* carriage in a randomized population-based study in Germany. Int. J. Infect. Dis..

[46] Martineau F., Picard F.J., Ke D., Paradis S., Roy P.H., Ouellette M., Bergeron M.G. (2001). Development of a PCR assay for identification of staphylococci at genus ans species levels. J. Clin. Microbiol..

[47] Cuny C., Abdelbary M., Layer F., Werner G., Witte W. (2015). Prevalence of the immune evasion gene cluster in *Staphylococcus aureus* CC398. Vet. Microbiol..

[48] Strommenger B., Kehrenberg C., Kettlitz C., Cuny C., Verspohl J., Witte W., Schwarz S. (2006). Molecular characterization of methicillin-resistant *Staphylococcus aureus* strains from pet animals and their relationship to human isolates. J. Antimicrob. Chemother..

[49] Cuny C., Layer F., Strommenger B., Witte W. (2011). Rare occurrence of methicillin-resistant Staphylococcus aureus CC130 with a novel mecA homologue in humans in Germany. PLoS ONE.

[50] Schlotter K., Ehricht R., Hotzel H., Monecke S., Pfeffer M., Donat K. (2012). Leukocidin genes lukF-P83 and *lukM* are associated with *Staphylococcus aureus* clonal complexes 151, 479 and 133 isolated from bovine udder infections in Thuringia, Germany. Vet. Res..

[51] Jarraud S., Mougel C., Thioulouse J., Lina G., Meugnier H., Forey F., Nesme X., Etienne J., Vandenesch F. (2002). Relationships between *Staphylococcus aureus* genetic background, virulence factors, agr groups (alleles), and human disease. Infect. Immun..

