# Supplementary material for: Nasal Colonization of Humans with Occupational Exposure to Raw Meat and to Raw Meat Products with Methicillin-Susceptible and Methicillin-Resistant Staphylococcus aureus
Source: Toxins (Basel). 2019 Mar 30;11(4):190. doi: 10.3390/toxins11040190 (PMC6521318; doi:10.3390/toxins11040190)
Supplement: Supplementary file 1 [file toxins-11-00190-s001.pdf]

Supplementary Materials: Nasal Colonization of Humans with Occupational Exposure to Raw Meat and to Raw Meat Products with Methicillin-Susceptible and Methicillin-Resistant *Staphylococcus aureus*

Christiane Cuny, Franziska Layer, Sonja Hansen, Guido Werner and Wolfgang Witte

Table 1. Prevalence and factors associated with nasal *S. aureus* carriage.

Characteristics		All	*S. aureus*+ (%)	*p*	Butchers, Meat Sellers	*S. aureus* + (%)	*p*	Cooks	*S. aureus* + (%)	*p*	Butcher vs. Cooks *p*
		605	130 (21.4%)		286	77 (26.90%)		319	53 (16.6%)		0.002
Sex	female	394	70 (17.8%)		195	44 (22.50%)		199	26 (13.1%)		0.019
	male	211	60 (28.4%)	0.03	91	33 (36.20%)	0.02	120	27 (22.5%)	0.04	0.013
Age	≤ 20	12	2		9	1		3	1		
	21–30	81	13 (16.0%)	0.26	33	9 (27.2%)	1.000	48	4 (8.3%)	0.19	0.07
	31–40	116	26 (22.4%)	0.50	41	14 (34.1%)	0.34	75	12 (16.0%)	0.04	0.04
	41–50	150	34 (22.6%)	0.77	80	22 (27.1%)	1.000	70	12 (17.1%)	0.13	0.18
	51–60	182	42 (23.0%)	0.61	92	24 (26.0%)	0.93	90	18 (20.0%)	0.32	0.42
	≥ 61	64	13 (20.3%)	0.08	31	7 (22.0%)	0.72	33	6 (18.1%)	0.76	0.89
Hospital stay	no	568	121 (21.3%)		278	76 (27.3%)		290	45 (15.5%)		
	yes	37	9 (24.3%)	0.67	8	1		29	8 (27.6%)	0.16	
Antibiotic prescript.	no	545	119 (21.8%)		264	73 (26.2%)		281	46 (16.3%)		
	yes	60	11 (18.3%)	0.64	22	4 (18.8%)	0.46	38	7 (18.4%)	0.93	
Diabetes mellitus	no	582	126 (21.7%)		274	74 (27.60%)		308	52 (16.8%)		
	yes	23	4	0.81	12	3	1.000	11	1	0.78	
Skin disorders	no	578	126 (21.8%)		267	75 (28.10%)		311	51 (16.3%)		
	yes	27	4 (14.8%)	0.53	19	2		8	2		
Pet animal contact	no	279	59 (21.1%)		110	28 (25.50%)		169	31 (18.3%)		
	yes	326	71 (21.8%)	0.45	176	49 (27.80%)	0.61	150	22 (14.7%)	0.456	< 0.001

Table S2. Distribution of spa-types and clonal complexes among *S. aureus* from butchers and meat sellers, and from cooks.

Clonal Complex	All No.	Isolates from Butchers/Meat Sellers No. *spa*-Types	Isolates from Cooks No. *spa*-Types
	**130**	**77**	**53**
CC1	3 (0.8%)	3 (4%) t127 (1)	
CC5	7 (5.4%)	5 (7%) t002 (3), t1265 (1), t1794 (1)	2 (3.8%) t002 (2)
CC7	16 (13.0%)	10 (13%) t091(9), t1943 (1)	6 (13.2%) t091 (5), t2932 (1)
CC8	13 (11.7%)	9 (12%) t008 (7), t121 (2),	4 (11.3%) t008 (4), t190 (1), t292 (1)
CC9	1 (0.8%)	1 (1.3%) t209 (1)	
CC15	25 (19.2%)	9 (15%) t084 (5), t346 (1), t499 (1) t15546 (1), t5497 (1)	16 (24.5%) t084 (9), t346 (3), t491 (1), t499 (1), t15664 (1), t15712 (1)
CC22	21 (14.8%)	15 (19%) t005 (8), t006 (1), t032 (2), t223 (1), t417 (1), t310 (1), t17201 (1)	6 (13.2%) t005 (4), t449 (1), t420 (1)
CC30	15 (11.7%)	11(14%) t012 (4), t018 (2), t021 (1), t122 (1), t253 (1), t789 (1) t1827(1)	4 (7.5%) t012 (2), t021 (1), t338 (1)
CC34	4 (3.1%)	2 (3%) t089 (1), t166 (1)	2 (3.7 %) t089 (1), t136 (1)
CC45	16 (12.5%)	8 (11%) t015 (4), t073 (2), t505 (1), t1460 (1)	8 (15%) t004 (1), t015 (3), t073 (2), t331 (1), t15726 (1)
ST101	2 (1.6%)	1 (1.3%) t056 (1)	1 (1.9%) t056 (1)
CC121	2 (1.6%)	1 (1.3%) t159 (1)	1 (1.9%) t159 (1)
CC182	1 (1%)	1 (1.3%) t493 (1)	
CC398	4 (3.1%)	1 (1.3%) t571 (1)	3 (5.7%) t571 (1), t1451 (2)

Table S3. Results from PCR for attribution to animal adapted subpopultions.

CC (n)	*Spa*-types (n)	IEC						SAPIAv	*luk*M	Seo131	Canonical SNP in SAPIG 2511	
		Int3	*sak*	*chp*	*scn*	*sea*	*sep*				C (human)	T (animal)
5 (7)	t002 (3)	+	+	+	+	-	-	-	n.d.	n.d.	n.d.	n.d.
	t002 (1)	+	+	-	+	-	-	-	n.d.	n.d.	n.d.	n.d.
	t002 (1)	+	+	-	-	+	-	-	n.d.	n.d.	n.d.	n.d.
	t1265 (1)	+	+	-	+	-	-	-	n.d.	n.d.	n.d.	n.d.
	t1794 (1)	+	+	-	+	-	-	-	n.d.	n.d.	n.d.	n.d.
7 (16)	t091 (5)	+	+	-	+	-	-	n.d.	n.d.	n.d.	n.d.	n.d.
	t091 (3)	+	+	-	-	+	+	n.d.	n.d.	n.d.	n.d.	n.d.
	t091 (1)	+	-	+	+	-	-	n.d.	n.d.	n.d.	n.d.	n.d.
	t091 (3)	+	+	+	+	-	-	n.d.	n.d.	n.d.	n.d.	n.d.
	t091 (3)	-	-	-	-	-	-	n.d.	n.d.	n.d.	n.d.	n.d.
	t2932 (1)	+	+	-	-	-	-	n.d.	n.d.	n.d.	n.d.	n.d.
8 (13)	t008 (1)	+	+	-	+	+	-	n.d.	-	-	n.d.	n.d.
	t008 (5)	+	+	-	+	-	-	n.d.	-	-	n.d.	n.d.
	t008 (3)	+	+	+	+	-	-	n.d.	-	-	n.d.	n.d.
	t121 (2)	+	+	-	+	-	-	n.d.	-	-	n.d.	n.d.
	t190 (1)	+	-	+	+	+	-	n.d.	-	-	n.d.	n.d.
	t292 (1)	+	+	-	+	-	-	n.d.	-	-	n.d.	n.d.
9 (1)	t209	+	+	+	+	-	-					
398 (4)	t571 (2)	+	-	+	+	-	-	n.d.	n.d.	n.d.	+	-
	t1451 (2)	+	+	+	+	-	-	n.d.	n.d.	n.d.	+	-

n.d. = not determined.
